# *Leishmania donovani* Transmission Cycle Associated with Human Infection, *Phlebotomus alexandri* Sand Flies, and Hare Blood Meals, Israel^1^

**DOI:** 10.3201/eid2905.221657

**Published:** 2023-05

**Authors:** Liora Studentsky, Laor Orshan, Fouad Akad, Irina Ben Avi, Debora Diaz, Shirly Elbaz, Orly Sagi, Gal Zagron, Lea Valinsky, Maya Davidovich-Cohen, Gad Baneth

**Affiliations:** Ministry of Health Central Laboratories for Public Health, Jerusalem, Israel (L. Studentsky, L. Orshan, I. Ben Avi, D. Diaz, S. Elbaz, M. Davidovich-Cohen);; The Hebrew University of Jerusalem, Rehovot, Israel (L. Studentsky, G. Baneth);; Ministry of Health National Laboratory for Public Health, Tel Aviv, Israel (F. Akad);; Soroka University Medical Center, Beer-Sheba, Israel (O. Sagi);; Israeli Ministry of Environmental Protection, Jerusalem (G. Zagron);; Maccabi Healthcare Services, Tel Aviv (L. Valinsky)

**Keywords:** cutaneous leishmaniasis, *Leishmania donovani*, *Leishmania infantum*, *Phlebotomus alexandri*, parasites, vector-borne infections, zoonoses, sand fly, high-resolution melting, HRM, Israel

## Abstract

Cutaneous leishmaniasis caused by *Leishmania major* or *L. tropica* and visceral leishmaniasis caused by *L. infantum* have been reported in Israel. We collected *Phlebotomus* spp. sand flies in the Negev desert of southern Israel to identify circulating *Leishmania* spp. Of 22,636 trapped sand flies, 80% were *P. alexandri*. We sequenced *Leishmania*-specific internal transcribed spacer 1 fragments and *K26* genes. Of 5,019 *Phlebotomus* female sand flies, 2.5% were *Leishmania* DNA–positive; 92% of infections were *L. donovani*. Phylogenetic analyses showed separate clustering of *L. donovani* and *L. infantum*. *P. alexandri* flies positive for *L. donovani* harbored blood meals from European hares. *Leishmania* DNA isolated from a patient with cutaneous leishmaniasis who lived in the survey area was identical to *L. donovani* from *P. alexandri* flies. We report circulation of *L. donovani*, a cause of visceral leishmaniasis, in southern Israel. Prompt diagnosis and *Leishmania* spp. identification are critical to prevent leishmaniasis progression.

Zoonotic leishmaniasis is endemic to Israel. *Leishmania* *tropica*, *L*. *major*, and *L*. *infantum* infect humans in different areas of Israel and circulate through distinct zoonotic transmission cycles (*1*). Cutaneous leishmaniasis (CL) is caused by *L*. *major*, which is transmitted by *Phlebotomus* *papatasi* sand flies, and *L*. *tropica*, which is transmitted by *P*. *sergenti* and *P*. *arabicus* sand flies. Canine leishmaniasis and human visceral leishmaniasis (VL) are caused by *L*. *infantum* in Israel, and the putative vectors are *P*. *perfiliewi*, *P*. *syriacus*, and *P*. *tobbi* sand flies (*1*–*9*). Reservoirs for *L*. *major* are sand rats (*Psammomys* *obesus*), gerbils (*Gerbillus* *dasyurus*), jirds (*Meriones* *crassus* and *M*. *tristrami*), and possibly also voles (*10*–*13*), whereas rock hyraxes (*Procavia* *capensis*) are considered the animal reservoir for *L*. *tropica* in Israel (*14*). Domestic dogs (*Canis* *lupus* *familiaris*), jackals (*C.* *aureus*), foxes (*Vulpes* *vulpes*), and wolves (*C.* *lupus*) are recognized reservoir hosts for *L*. *infantum* (*15*).

A substantial increase in CL incidence has been recorded since 2002, and endemic transmission has occurred in areas of Israel where it was previously unknown (*6**,**16**,**17*). Although not life-threatening, CL is a considerable public health problem in Israel; CL is diagnosed in hundreds of new patients annually. During 2001–2018, CL incidence rates increased 7-fold, from 0.4 to 2.9/100,000 population; a peak was observed in 2012, when the mean annual incidence increased to 4.4/100,000 population (*18*,*19*). Our study combines results from sand fly surveys, *Phlebotomus* spp. blood meal analysis, and human patient clinical data from the mountainous area of central Negev in southern Israel during the summer months of 2018–2020. We found a fourth leishmaniasis transmission cycle associated with human illness.

## Materials and Methods

### Study Area and Sand Fly Trapping

**Figure 1:**
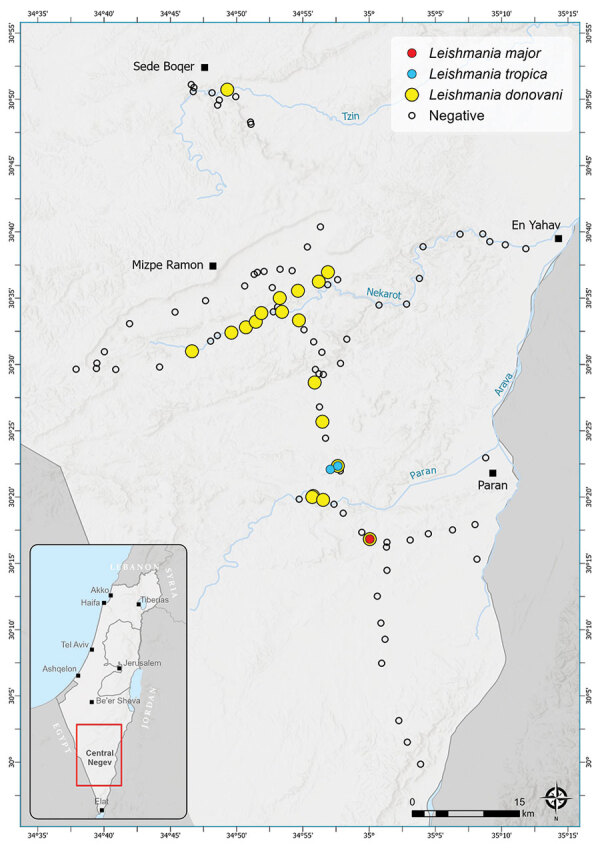
Locations of *Phlebotomus* spp. sand fly collection sites within the central Negev region of Israel in study of *Leishmania donovani* transmission cycle associated with human infection, *Phlebotomus alexandri* sand flies, and hare blood meals. We collected sand flies outdoors in August 2018, September 2019, and August 2020 by using modified traps from the US Centers for Disease Control and Prevention. The traps operated without light and were powered by 2 AA (1.2V) rechargeable batteries and baited with ≈1 kg dry ice. Traps were placed in an updraft vertical position overnight; openings were ≈10 cm above the ground, and collection cups hung above the motor and fan. Different colored circles indicate sites where specific *Leishmania* spp. infections were identified in trapped *Phlebotomus* sand flies. Empty circles indicate sites where sand flies were negative for *Leishmania* spp. Inset shows location of the survey area in Israel (red box).

We conducted our study in the mountainous desert area of central Negev in southern Israel (Figure 1). In this region, elevations range from 50 to 1,037 m above sea level, large differences occur between peak daytime and nighttime temperatures, and annual average precipitation is 30–150 mm (*20*,*21*). We collected sand flies outdoors in August 2018, September 2019, and August 2020 by using modified traps from the US Centers for Disease Control and Prevention. The traps operated without light and were powered by 2 AA (1.2V) rechargeable batteries and baited with ≈1 kg dry ice. We placed traps in an updraft vertical position overnight; openings were ≈10 cm above the ground, and collection cups hung above the motor and fan (*22*,*23*). 

### Identification and Sample Preparation

We transferred live sand fly catches to the laboratory, which we then chilled and processed. We counted dead sand flies and sorted by sex, identifying all male flies at the species level by using specific morphologic keys for genitalia (*24*,*25*). We kept all engorged females and <10–15 unfed females from each trap individually. If the number of female sand flies in the trap was >15, we pooled those flies with others in groups of 20 specimens each. We noted the blood meal size and freshness for each engorged female (*26*). We stored all female fly specimens in collection microtubes at −80°C until DNA extraction.

### Molecular Analysis by Real-Time PCR, HRM Assay, and Sequencing

We extracted total DNA from sand fly samples by using the QIAsymphony DSP DNA Mini Kit and QIAsymphony SP robot (QIAGEN, https://www.qiagen.com). We homogenized the samples for 5 min in 50 μL lysis buffer and stainless steel beads by using a TissueLyser II instrument (QIAGEN). The lysis buffer contained DNase- and proteinase-free RNaseA (ThermoFisher Scientific, https://www.thermofisher.com), proteinase K, and ATL tissue lysis buffer (QIAGEN). After homogenization, we added 200 μL lysis buffer to each samples and incubated at 56°C for 2 h. We performed centrifugation and transferred the samples directly to the robot. We extracted DNA in accordance with the manufacturer’s instructions and eluted the DNA in 100 μL of elution buffer.

We performed all real-time PCR reactions by using a Roche LightCycler 96 (Roche, https://www.roche.com) and AccuMelt HRM SuperMix (Quantabio, https://www.quantabio.com). We analyzed all female sand flies for *Leishmania* spp. infection and single and engorged female flies to determine *Phlebotomus* sand fly species and blood meal source. We performed high-resolution melting (HRM) assays at the final step of each real-time PCR. We performed amplicon dissociation analysis by capturing fluorescence signals in 0.1°C/s increments and holding for 60 s in each range of the melting curve (60°C–85°C for sand fly species and blood meal detection assays or <95°C for *Leishmania* PCR). Sanger sequencing was performed at the Center for Genomic Technologies at Hebrew University of Jerusalem.

We screened all female sand flies for *Leishmania* DNA and identified parasite species by amplifying an internal transcribed spacer (ITS) 1 rRNA fragment with ITS1–219 PCR primers (Appendix Table 1) and by using the HRM assay (*27*). For PCR controls, we extracted DNA from parasite promastigote cultures of international reference strains: *L. major* (MHOM/PS/1967/JerichoII), *L. tropica* (MHOM/IL/1990/P283), *L. infantum* (MHOM/SD/62/2S), *L. donovani* (MHOM/SD/1962/1S-CLD2), and *L. aethiopica* (MHOM/ET/1972/L102). High purity water for molecular biology (Bio-Lab, http://www.biolab-chemicals.com) was used as a negative control.

**Table 1:** Human clinical samples from Soroka Medical Center in study of *Leishmania donovani* transmission cycle associated with human infection, *Phlebotomus alexandri* sand flies, and hare blood meals, Israel*

Infecting *Leishmania* sp.†	Residence	Clinical description	Age, y/sex	Diagnosis	Patient no.
*L. infantum* (100% identity with *L. infantum*, GenBank accession no. KU680954)	Northern Israel	Splenomegaly	47/M	VL	1
*L. infantum* (99.19% identity with *L. infantum*, GenBank accession no. MN503527)	Hebron	Splenomegaly, hepatomegaly	4/F	VL	2
*L. infantum* (99.87% identity with *L. infantum*, GenBank accession no. KU680954)	Negev	Skin ulcer	69/F	CL	3
*L. donovani* (99.75% identity with *L. donovani*, GenBank accession no. LC459330)	Arava	Skin ulcer	51/M	CL	4

*CL, cutaneous leishmaniasis; VL, visceral leishmaniasis. †*Leishmania* spp. were identified by PCR high resolution melting curves and Sanger sequencing.

We included DNA isolated from skin lesions from 4 patients who had leishmaniasis diagnosed at the parasitology laboratory at Soroka Medical Center, Beer-Sheba, Israel; leishmaniasis was caused by *L. donovani/L. infantum* complex in those patients (Table 1). Leishmaniasis was diagnosed at the hospital by using multiplex real-time PCR with 5 probes for the ITS region of *Leishmania* sp. (*28*,*29*). We analyzed the samples further at the Ministry of Health by using real-time PCR–HRM amplification of the ITS1 fragment and ITS region and then sequencing.

We amplified the entire 1,020-bp ribosomal ITS region from *Leishmania*-positive field, clinical, and control samples by using PCR primers LITSR and LITSV (Appendix Table 1). If the entire ITS region was not successfully amplified with LITSR and LITSV primers, we used an internal pair of primers, L5.8S and L5.8SR, to amplify ITS1 and ITS2 separately (*30*). We performed ITS1 amplicon sequencing for 12 of the positive samples, and the entire ITS region was sequenced from 6 unfed and 3 engorged females, 3 pooled *Phlebotomus* spp. samples, all 4 human samples, and 4 *Leishmania*-positive controls. We amplified the repeat region of the *L. donovani* and *L. infantum* *HASPB* (known as *K26*) gene for additional separation of *L. donovani* complex–positive samples by using primers K26F and K26R (*31*).

To identify *Phlebotomus* spp., we amplified a 368–393-bp fragment of the cytochrome b gene by using a universal primer set designed for this study (cytb-F and cytb-R; Appendix Table 1). The specificity of the designed primers was tested against DNA sequences from hematophagous arthropods, including sand flies, mosquitoes, and ticks. Male sand flies identified at the species level by using morphologic characteristics were used as positive controls and molecular biology grade water was used as a negative control. We analyzed all individual samples, and 1 third of samples from each melting curve pattern were sequenced.

We identified blood meal sources in *Phlebotomus* sand fly specimens by amplifying a 500-bp segment of host 12S and 16S mitochondrial rRNA genes by using modified vertebrate universal primers N12–16F and N12–16R (*32*). We included negative (water) and positive (100 ng of human DNA) controls in each PCR. We sequenced 50 samples that represented all HRM curve patterns and all female sand flies containing blood meals that had a melting curve of a rare host (<5 samples).

We used DNA from *Leishmania* reference strains, male sand fly specimens identified by morphologic characteristics, and human blood as templates for real-time PCR and HRM curve standardization. Each species produced a unique melting curve that was easily distinguishable from other species and consistent with observed nucleotide differences (Appendix Figures 1–3). We compared normalized HRM curves of field samples with the positive control included in each PCR, which enabled species determination (*27*). We validated species identification by sequencing 1 third of the samples; complete matches were observed for speciation by HRM curve analysis and DNA sequencing.

**Figure 3:**
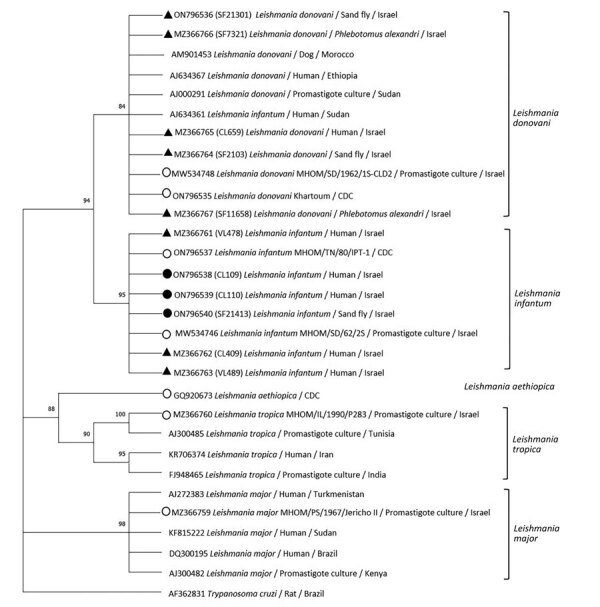
Phylogenetic analysis of entire *Leishmania* internal transcribed spacer region in study of *Leishmania donovani* transmission cycle associated with human infection, *Phlebotomus alexandri* sand flies, and hare blood meals, Israel. *Leishmania*-specific internal transcribed spacer region (988 bp) was amplified by PCR from *P. alexandri* sand flies, pooled female *Phlebotomus* spp. flies, and patient samples and then sequenced. Tree was constructed by using by using the maximum-likelihood method and Tamura 3-parameter model of all relevant *Leishmania* spp. and *Trypanosoma* *cruzi* as an outgroup. Sand fly and clinical samples from this study (black triangles), *L. infantum* isolates from Israel (black circles), *Leishmania* international reference strains (empty circles), and available GenBank *Leishmania* sequences are shown. GenBank accession numbers, isolate source, and country of origin are shown for each sequence. Only bootstrap values >70% are shown next to branches. Not to scale.

We aligned and corrected nucleotide sequences by using BioNumerics version 8.0 software (Applied Maths, https://www.applied-maths.com) and compared sequences against the GenBank database by using BLASTN (http://blast.ncbi.nlm.nih.gov). We identified *Leishmania* spp., blood meal sources, and sand fly species on the basis of >98% identity with sequences obtained during the BLAST search. We submitted sequences of the ITS1 fragments and entire ITS and *K26* regions obtained in this study to GenBank (Appendix Table 2).

**Table 2:** Number of female and male *Phlebotomus* spp. collected during 2018–2020 in central Negev in study of *Leishmania* *donovani* transmission cycle associated with human infection, *Phlebotomus* *alexandri* sand flies, and hare blood meals, Israel

Phlebotomus spp.	2018		2019		2020		Total no. (%)
	F	M	F	M	F	M	
*P. alexandri*	129	1,452	70	126	431	4,039	6,247 (80.0)
*P. kazeruni*	38	837	22	11	78	142	1,128 (14.5)
*P. sergenti*	5	109	26	9	34	56	239 (3.1)
*P. papatasi*	25	97	8	8	8	27	173 (2.2)
*P. syriacus*	0	0	3	0	17	3	23 (0.3)
Not identified*	8,344	0	265	0	6,217	0	14,826
Total	8,541	2,495	394	154	6,785	4,267	22,636

*Sand flies were pooled before molecular testing in batches of 20 speciments each.

**Figure 2:**
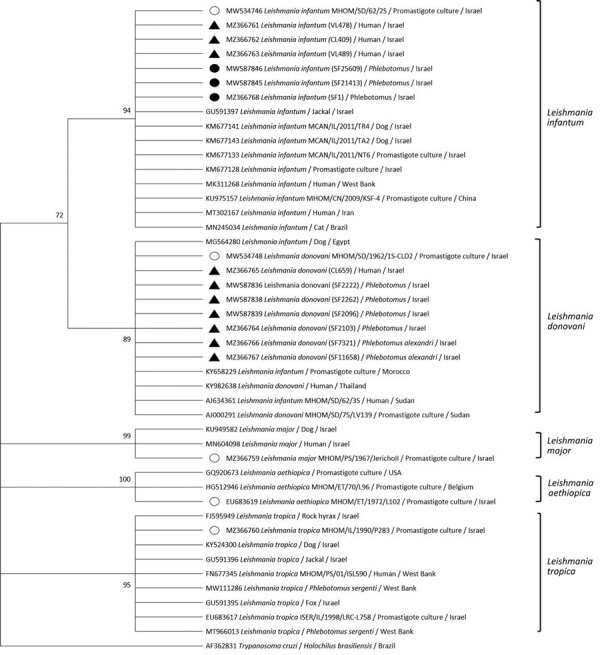
Phylogenetic analysis of *Leishmania* internal transcribed spacer 1 rRNA fragments in study of *Leishmania donovani* transmission cycle associated with human infection, *Phlebotomus alexandri* sand flies, and hare blood meals, Israel. *Leishmania*-specific internal transcribed spacer 1 rRNA fragments (201 bp) were amplified by PCR from *P. alexandri* sand flies, pooled female *Phlebotomus* spp. flies, and patient samples and then sequenced. Tree was constructed by using the maximum-likelihood method and Tamura 3-parameter model, estimated by using the Aikaike information criterion (*33*). Dendogram includes sequences from *L. donovani* and *L. infantum* isolated from sand flies and clinical samples in this study compared with *Leishmania* spp. reference controls and GenBank sequences from Israel and other countries. Tree shows substantial separate clustering of *L. infantum* (boostrap 94%) and *L. donovani* (bootstrap 89%) sequences. Empty circles are *Leishmania* international reference strains, black triangles are the 10 sequences from our study deposited in GenBank, and black circles are additional *L. infantum*–positive sand flies samples from Israel. Available GenBank sequences for *L. major, L. tropica*, *L. infantum*, and *L. donovani* from Israel and other countries are also included. GenBank accession numbers, *Leishmania* spp., isolate source, and country are indicated. Only bootstrap values >70% are shown. Not to scale.

**Figure 4:**
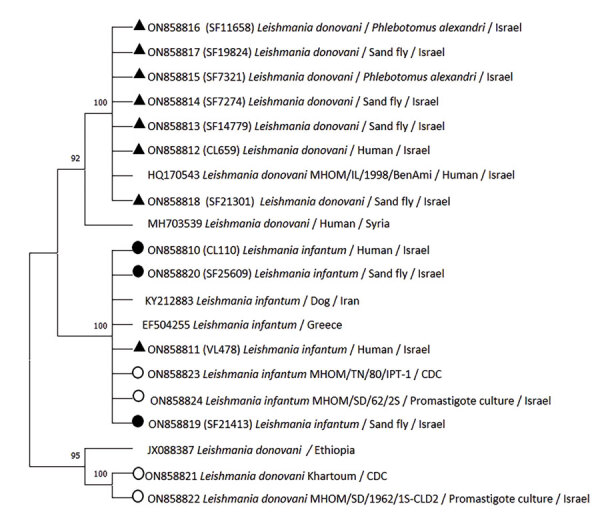
Phylogenetic analysis of *Leishmania* *K26* gene in study of *Leishmania donovani* transmission cycle associated with human infection, *Phlebotomus alexandri* sand flies, and hare blood meals, Israel. *Leishmania*-specific *K26* gene fragment (348 bp) was amplified by PCR from *P. alexandri* flies, pooled female *Phlebotomus* spp. flies, and patient samples and then sequenced. Tree was constructed by using the maximum-likelihood method and Hasegawa-Kishino-Yano model. *K26* phylogenetic analysis shows separation between *L. infantum* and *L. donovani*. Sand fly and clinical samples from this study (black triangles), *L. infantum* isolates from Israel (black circles), *Leishmania* international reference strains (empty circles), and available GenBank *Leishmania* sequences are shown. GenBank accession number, isolate source, and country of origin are shown for each sequence. Only bootstrap values >70% are shown next to branches. Not to scale.

We constructed phylogenetic trees on the basis of marker gene sequences in this study and relevant sequences of other *Leishmania* spp. deposited in GenBank. We used MEGA X software (*33*) to infer phylogenetic trees after nucleotide sequence alignment was performed by using ClustalW software (http://www.clustal.org) and maximum-likelihood and neighbor-joining algorithms. We used 1,000 bootstrap replicates to determine percentages of replicate trees. We constructed a phylogenetic tree composed of 45 analyzed partial sequences of the ITS1 locus, including sequences of *Leishmania* spp. from Israel and other countries deposited in GenBank and *Trypanosoma cruzi* as an outgroup (Figure 2). We constructed a second tree that included 30 nearly complete ITS sequences of all relevant *Leishmania* spp. and *T. cruzi* as an outgroup (Figure 3) and an additional phylogenetic tree that included 20 *K26* gene sequences of *L. donovani* complex–positive samples (Figure 4).

## Results

We collected 22,636 *Phlebotomus* spp. sand fly specimens (15,720 female and 6,916 male; sex ratio 2.3) during 7 trapping nights by using 118 traps placed at 94 sites. After identifying all male and 894 (6%) female flies, we found the catches consisted of 5 species. The most abundant sand fly species were *P*. *alexandri* (80%), *P*. *kazeruni* (14.4%), *P*. *sergenti* (3.1%), *P*. *papatasi* (2.2%), and *P*. *syriacus* (0.3%) (Table 2).

Among the 4,140 unfed female sand flies tested in 210 pools,we found 41 pools were positive for *Leishmania* spp. In addition, 6/688 single female flies and 4/206 engorged female flies were positive for *Leishmania* spp. Of the 51 *Leishmania*-positive samples, the HRM curves of 47 (36 pools, 6 single females, and 3 engorged female flies) were similar to the HRM curve of the *L. donovani* control (Table 3; Appendix Figure 1). The HRM curves for 2 pooled fly samples from 2018 were identical to the HRM curve of the *L. tropica* control. One pooled fly sample and 1 engorged female fly collected in 2020 had an HRM curve identical to the *L. major* control. The ITS1–PCR sequences of 20 samples (11 pools and all 9 single and engorged females) that had HRM curves similar to the *L. donovani* control HRM curve were also 100% identical to the *L. donovani* control sequence.

**Table 3:** Number of *Leishmania* spp. detected in sand fly samples by PCR during 2018–2020 in study of *Leishmania donovani* transmission cycle associated with human infection, *Phlebotomus alexandri* sand flies, and hare blood meals, Israel*

Year	Total no. tested	*L*. *donovani*	*L*. *tropica*	*L*. *major*
2018				
Females (no. pools)	2,938 (148)	24	2	0
Single unfed females	108	0	0	0
Single engorged females	89	0	0	0
2019				
Females (no. pools)	262 (15)	4	0	0
Single unfed females	121	3	0	0
Single engorged females	8	0	0	0
2020				
Females (no. pools)	940 (47)	10	0	1
Single unfed females	459	3	0	0
Single engorged females	109	3	0	1
Total no.	5,019	47	2	2

*All engorged females and <10–15 unfed females from each trap were maintained and analyzed individually. If the number of female sand flies in the trap was >15, they were pooled in batches of 20 specimens each.

Leishmaniasis was diagnosed in 4 human patients (Table 1). The ITS1 HRM curve and sequence from patient 4 with CL were similar to the *L. donovani* control and 47 *L*. *donovani–*positive *Phlebotomus* spp. samples. The ITS1 HRM and sequences from patients 1 and 2 with VL and patient 3 with CL were similar to the *L. infantum* control. Alignment of ITS1 sequences from *L. infantum* and *L. donovani* controls, 3 representative sand fly samples showing HRM identical to *L. donovani*, and the 4 patient samples showed clustering into 2 distinct groups. The first group comprised the *L. donovani* control, 3 sand fly samples, and patient 4. The second group comprised the *L. infantum* control and samples from patients 1, 2, and 3. The difference between the groups was at position 71–74 in ITS1; the *L. donovani* group had a 4-nt (ATAT) insertion that was missing in the *L. infantum*–positive samples. A comparison of ITS1 sequences with those in GenBank showed 100% query coverage and 99.65%–100% identity with GenBank sequences for *L. infantum* and *L. donovani* from various countries (data not shown).

We aligned DNA sequences from the entire ITS region obtained from *Leishmania*–positive *P. alexandri* samples from our study and the *L. infantum* and *L. donovani* controls (Appendix Figure 4). We found 2 additional regions containing polymorphic sites that distinguished between *L. infantum* and *L. donovani*: a 2-nt (GG) deletion at position 724–725 and 1-nt (G) insertion at position 817 in the *L. donovani* sequence.

We constructed a phylogenetic tree of ITS1 rRNA fragments of *Leishmania* sequences obtained from *P. alexandri* flies, pooled female *Phlebotomus* spp. flies, and patient samples from this study. We compared those sequences with *Leishmania* spp. controls and GenBank sequences from Israel and other countries. The tree showed substantial separate clustering of *L. infantum* (boostrap 94%) and *L. donovani* (bootstrap 89%) sequences (Figure 2). Phylogenetic analysis of the entire ITS region showed separate clustering of *L. infantum* (bootstrap 84%) and *L. donovani* (bootstrap 95%) sequences (Figure 3). *K26* phylogenetic analysis also showed separation between *L. infantum* and *L. donovani* (Figure 4).

We identified the blood meal source for 182/206 (88%) engorged female sand flies that represented the 4 most abundant sand fly species within the study area. We observed 7 types of HRM curves. We compared blood meal sequences with GenBank sequences and determined similarities between HRM curves. We identified European brown hare (*Lepus europaeus*) blood in 126 (69.2%) flies, onager (*Equus hemionus*) blood in 33 (18.3%) flies, gazelle (*Gazella dorcas*) blood in 16 (8.8%) flies, and domestic dog (*C. lupus familiaris*) blood in 4 (2.2%) flies; 1 female sand fly each contained blood from either a fat sand rat (*Psammomys obesus*), fox (*V. vulpes*), or human (Table 4). Hare blood was the dominant blood meal found in all 4 *Phlebotomus* spp. flies: *P. papatasi*, 38%; *P. sergenti*, 67%; *P. alexandri*, 71%; and *P. kazeruni*, 89%. The 12S–16S hare blood meal sequences were 99.8% similar to *L. europaeus* hares and only 95.3% similar to *L. capensis* hares.

**Table 4:** Number of female *Phlebotomus* spp. sand flies collected in the central Negev region engorged with different blood meals in study of *Leishmania donovani* transmission cycle associated with human infection, *Phlebotomus alexandri* sand flies, and hare blood meals, Israel

Blood meal source	*Phlebotomus* spp.				Total no.
	*P*. *alexandri*	*P*. *kazeruni*	*P*. *papatasi*	*P*. *sergenti*	
*Lepus europaeus* (hare)	107	8	5	6	126
*Equus hemionus* (onager)	31	1	0	1	33
*Gazella dorcas* (gazelle)	10	0	4	2	16
*Canis lupus familiaris* (dog)	1	0	3	0	4
*Homo sapiens* (human)	1	0	0	0	1
*Psammomys obesus* (fat sand rat)	0	0	1	0	1
*Vulpes vulpes* (fox)	1	0	0	0	1
Total no.	151	9	13	9	182

Of the 47 *Phlebotomus* spp. sand fly samples with HRM curve patterns and sequences similar to the *L. donovani* control, 9 were single *P. alexandri* female sand flies, 3 of which were engorged with hare blood. Of the 2 *Phlebotomus* spp. samples positive for *L. major*, 1 was in a single engorged *P. papatasi* female sand fly that had an unsuccessful blood meal identification. The 2 identified *L. tropica* samples were from pooled female sand flies.

## Discussion

We found a fourth transmission cycle of leishmaniasis in the central Negev region of southern Israel. On the basis of molecular analysis of the ITS region and *K26* gene and phylogenetic analysis, we concluded that the parasite found in patient 4, who lives in the survey area, and in *P. alexandri* sand flies was *L. donovani* sensu stricto. We found that *L. infantum* was the cause of illness in the other 3 patients with leishmaniasis. A case report describing patient 4 was published in 2016; the authors concluded that the infecting parasite was likely *L. infantum* because of prevailing knowledge of endemic *Leishmania* transmission in Israel (*29*). An earlier study reported another patient from the Arava region of central Negev, close to where patient 4 lives, who had symptoms of both CL and VL (*34*). The cause of infection was identified as *L. donovani*; the authors noted that this infection was unusual because *L. donovani* was not known to circulate in Israel. The earlier study substantiates our findings of *L. donovani* in both sand flies and another human patient within the same geographic area. *L. infantum* was identified as the causative agent in the other 3 patients in our study and was also described in canines in Israel (*1*).

The high abundance of *P. alexandri* sand flies within the study area and the association with *L. donovani* infections suggest that the *P. alexandri* sand fly is the putative vector of *L. donovani* in Israel. *P*. *alexandri* flies have been associated with *L. donovani* sensu latu transmission in other parts of the Old World. Natural infection by *L. donovani* was found in field-collected *P. alexandri* sand fly specimens in China, and inoculation of hamsters with those parasites caused VL (*35*). Another study reported the susceptibility of *P. alexandri* to artificial infection with *L. donovani* isolated from human patients in China (*36*).

We found blood meals from European brown hares in ≈70% of engorged female *Phlebotomus* sand flies in our study. The high feeding rates on hares, presence of *L. donovani* in female *P. alexandri* sand flies engorged with hare blood and illnesses reported in humans infected with *L. donovani* suggest a zoonotic *L. donovani* transmission cycle in Israel. Those data suggest that the hare could be a potential reservoir and *P. alexandri* flies could be the putative vector for *L. donovani*. The role of hares as a reservoir host for *L.* *donovani* requires further investigation; however, a related hare species, *Lepus granatensis*, was reported as a potential sylvatic reservoir for *L. infantum* in a leishmaniasis outbreak in Madrid, Spain (*37*,*38*). Furthermore, studies in Greece and Italy detected *L. donovani* complex infection in *L. europaeus* hares (*39*,*40*), providing support for hares as a potential reservoir for *L.* *donovani* in Israel. Dogs were identified as reservoirs for *L. donovani* in India, Sudan, and Ethiopia, and different rodent species have been identified as possible reservoirs of *Leishmania* spp. from the *L*. *donovani* complex (*41*–*48*). However, no *L. donovani* infections in canines and rodents have been reported in Israel; infections in sand fly blood meals found in our study do not implicate those hosts in the local life cycle of *L. donovani*.

In conclusion, we found circulation of *L. donovani* in the Negev region of southern Israel that was associated with cutaneous lesions in humans. We determined that *P. alexandri* was the putative sand fly vector and that hares were the main reservoir host of *L. donovani*. We found 2 distinct *Leishmania* spp. in the *L. donovani* complex in Israel. Previously, the few reported human cases of CL resulting from *L. donovani* infections were attributed to either *L. infantum* or nonautochthonous infections. Analysis of patient samples in our study indicates that, in addition to *L. major* and *L. tropica* (the known agents causing CL), *L. donovani* is also a cause of autochthonous CL in Israel. Our results suggest that CL in Israel can be caused by *L. donovani*, a primary cause of VL. Therefore, prompt diagnosis, identification of the *Leishmania* sp., and treatment with drugs intended for visceral leishmaniasis, such as pentavalent antimonials or liposomal amphotericin B (*49*), are critical to prevent disease progression and death among patients with leishmaniasis.

Appendix Additional information for *Leishmania donovani* transmission cycle associated with human infection, *Phlebotomus alexandri* sand flies, and hare blood meals, Israel.
