# Supplementary material for: Leishmania donovani Transmission Cycle Associated with Human Infection, Phlebotomus alexandri Sand Flies, and Hare Blood Meals, Israel
Source: Emerg Infect Dis. 2023 May;29(5):945–55. doi: 10.3201/eid2905.221657 (PMC10124660; doi:10.3201/eid2905.221657)
Supplement: Appendix — Additional information for Leishmania donovani transmission cycle associated with human infection, Phlebotomus alexandri sand flies, and hare blood meals, Israel. [file 22-1657-Techapp-s1.pdf]

Article DOI: https://doi.org/10.3201/eid2905.221657
EID cannot ensure accessibility for supplementary materials supplied by authors. Readers who have difficulty accessing supplementary content should contact the authors for assistance.

Leishmania donovani Transmission Cycle Associated with Human Infection, *Phlebotomus alexandri* Sand Flies, and Hare Blood Meals, Israel

Appendix

Appendix Table 1. Real-time PCR primers and protocols used for the detection and identification of *Leishmania* species, blood meal sources, and sand fly species*

Target gene	Primer	Amplicon, bp	Reference† or PCR protocol
ITS1	ITS1–219F: AGCTGGATCATTTTCCGATG<br>ITS1–219R: ATCGCGACACGTTATGTGAG	265	(*27*)
ITS	LITSR: CTGGATCATTTMCGATG<br>LITSV: ACACTCAGGTCTGTAAAC	1020	(*30*)
ITS1	LITSR: CTGGATCATTTMCGATG<br>L5.8S: TGATACCACTTATCGCACTT	320	
ITS2	L5.8SR: AAGTGCGATAAGTGGTA<br>LITSV: ACACTCAGGTCTGTAAAC	700	
K26	K26F: ACGAAGGACTCCRCAAAG<br>K26R: TTCCCATCGTTTTGCTG	350	(*31*)
cytb‡	cytb-F: GGAGGAGTAATYGCHYTTGTWATATC<br>cytb-R: AAGATATTTACCYGCTTCKTTATGTT	368–393	95°C, 5 min; then 45 cycles of 95°C, 5 s; 38°C, 2 s; 60°C, 45 s; 95°C, 60 s; 40°C, 60 s; 65°C, 1 s; 85°C, 1 s; 37°C, 30 s
12S, 16S	N12–16F: ACAYACCGCCCGTCACCCTC<br>N12–16R: AACCAGCTATCACMAGGCTCG	500 bp	(*32*)

*ITS, internal transcribed spacer, entire region; ITS1, internal transcribed spacer 1; ITS2, internal transcribed spacer 2; 12S, 16S, mitochondrial rRNA gene; cytb, cytochrome b gene.
†References are from the main text.
‡Primers designed for our study.

Appendix Table 2. Details of the samples from this study submitted to GenBank*

Accession no.†	*Leishmania* sp.	Isolate	Source	Locus	Size, bp
MW587834	*Leishmania donovani*	SF2101	*Phlebotomus*	ITS1	200
MW587835	*Leishmania donovani*	SF2103	*Phlebotomus*	ITS1	228
MW587836	*Leishmania donovani*	SF2222	*Phlebotomus*	ITS1	287
MW587837	*Leishmania donovani*	SF2226	*Phlebotomus*	ITS1	170
MW587838	*Leishmania donovani*	SF2262	*Phlebotomus*	ITS1	290
MW587839	*Leishmania donovani*	SF2096	*Phlebotomus*	ITS1	287
MW587841	*Leishmania donovani*	CL659 (#4)	Human	ITS1	232
MW587842	*Leishmania infantum*	CL409 (#3)	Human	ITS1	227
MW587843	*Leishmania infantum*	VL478 (#1)	Human	ITS1	246
MW587844	*Leishmania infantum*	VL489 (#2)	Human	ITS1	245
MW587845	*Leishmania infantum*	SF21413	*Phlebotomus*	ITS1	312
MW587846	*Leishmania infantum*	SF25609	*Phlebotomus*	ITS1	198
MW534746	*Leishmania infantum*	MHOM/SD/62/2S	Promastigote culture	ITS	1,024
MW534748	*Leishmania donovani*	MHOM/SD/1962/1S-CLD2	Promastigote culture	ITS	980
MZ366759	*Leishmania major*	MHOM/PS/1967/JerichoII	Promastigote culture	ITS	815
MZ366760	*Leishmania tropica*	MHOM/IL/1990/P283	Promastigote culture	ITS	783
MZ366761	*Leishmania infantum*	VL478 (#1)	Human	ITS	782
MZ366762	*Leishmania infantum*	CL409 (#3)	Human	ITS	783
MZ366763	*Leishmania infantum*	VL489 (#2)	Human	ITS	782
MZ366764	*Leishmania donovani*	SF2103	*Phlebotomus*	ITS	782

Accession no.†	*Leishmania* sp.	Isolate	Source	Locus	Size, bp
MZ366765	*Leishmania donovani*	CL659 (#4)	Human	ITS	787
MZ366766	*Leishmania donovani*	SF7321	*P. alexandri*	ITS	786
MZ366767	*Leishmania donovani*	SF11658	*P. alexandri*	ITS	785
ON796535	*Leishmania donovani*	Khartoum	ATCC	ITS	967
ON796536	*Leishmania donovani*	SF21301	*Phlebotomus*	ITS	985
ON796537	*Leishmania infantum*	MHOM/TN/80/IPT-1	ATCC	ITS	985
ON796538	*Leishmania infantum*	CL109	Human	ITS	1,029
ON796539	*Leishmania infantum*	CL110	Human	ITS	1,018
ON796540	*Leishmania infantum*	SF21413	*Phlebotomus*	ITS	712
ON858810	*Leishmania infantum*	CL110	Human	k26	766
ON858811	*Leishmania infantum*	VL478	Human	k26	620
ON858812	*Leishmania donovani*	CL659	Human	k26	360
ON858813	*Leishmania donovani*	SF14779	*Phlebotomus*	k26	361
ON858814	*Leishmania donovani*	SF7274	*Phlebotomus*	k26	367
ON858815	*Leishmania donovani*	SF7321	*P. alexandri*	k26	370
ON858816	*Leishmania donovani*	SF11658	*P. alexandri*	k26	365
ON858817	*Leishmania donovani*	SF19824	*Phlebotomus*	k26	371
ON858818	*Leishmania donovani*	SF21301	*Phlebotomus*	k26	362
ON858819	*Leishmania infantum*	SF21413	*Phlebotomus*	k26	504
ON858820	*Leishmania infantum*	SF25609	*Phlebotomus*	k26	478
ON858821	*Leishmania donovani*	Khartoum	ATCC	k26	278
ON858822	*Leishmania donovani*	MHOM/SD/1962/1S-CLD2	Promastigote culture	k26	278
ON858823	*Leishmania infantum*	MHOM/TN/80/IPT-1	ATCC	k26	619
ON858824	*Leishmania infantum*	MHOM/SD/62/2S	Promastigote culture	k26	616

*ITS, internal transcribed spacer, entire region; ITS1, internal transcribed spacer 1.
†GenBank accession no.

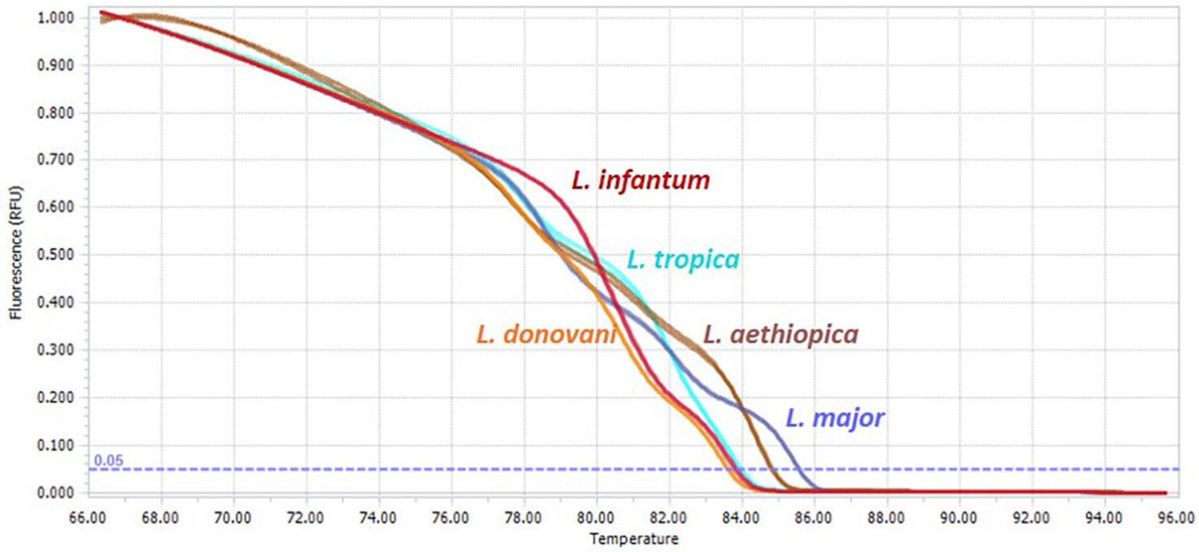

Appendix Figure 1. Normalized high resolution melting curves of 370-bp cytochrome b PCR amplicons from different *Leishmania* spp. isolated from sand flies trapped in the central Negev region, Israel. Normalized fluorescence is plotted against temperature.

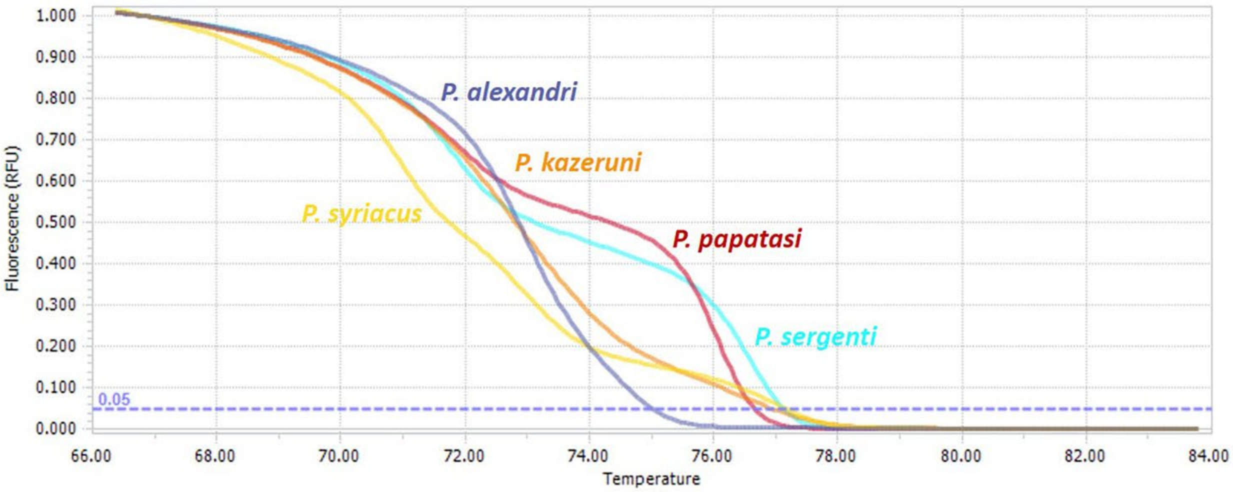

Appendix Figure 2. Normalized high resolution melting curves of *Leishmania*-specific internal transcribed spacer 1 rRNA fragment PCR amplicons isolated from different *Phlebotomus* spp. sand flies trapped in the central Negev region, Israel.

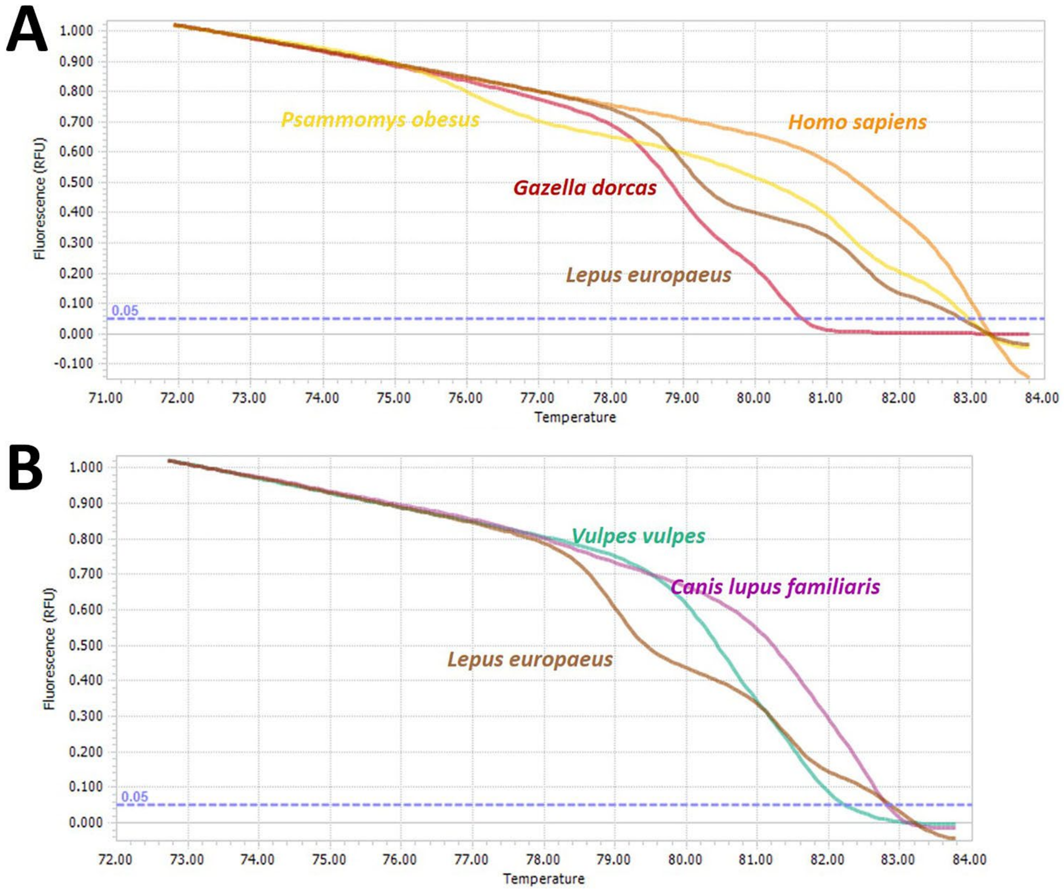

Appendix Figure 3. Normalized high resolution melting curves of blood meal PCR amplicons from different animal sources found in engorged sand fly females trapped in the central Negev region, Israel.

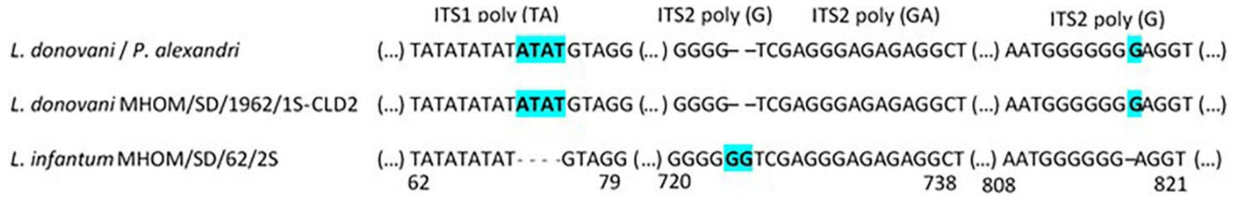

Appendix Figure 4. Alignment of sequences from the entire internal transcribed spacer region of the *18S* gene from *Leishmania donovani* found in *Phlebotomus alexandri* sand flies in the central Negev, Israel, and *L. donovani* and *L. infantum* international reference strains. Nucleotides marked in blue represent insertions, and discontinuous lines represent absence of nucleotides in sequences. Numbers correspond to nucleotide sequence position in the region. ITS1, internal transcribed spacer 1; ITS2, internal transcribed spacer 2.
